# Dosimetric robustness against setup errors in charged particle radiotherapy of skull base tumors

**DOI:** 10.1186/s13014-014-0279-2

**Published:** 2014-12-05

**Authors:** Filippo Ammazzalorso, Urszula Jelen, Rita Engenhart-Cabillic, Wolfgang Schlegel

**Affiliations:** Department of Radiotherapy and Radiation Oncology, University of Marburg, Baldingerstraße, Marburg, 35043 Germany; Department of Medical Physics in Radiation Oncology, German Cancer Research Center (DKFZ), Im Neuenheimer Feld 280, Heidelberg, 69120 Germany

**Keywords:** Dosimetric robustness, Setup errors, Beam setup optimization, Ion beam therapy, Proton therapy, Carbon ion therapy, Skull base tumors

## Abstract

**Background:**

It is expected that physical dose deposition properties render charged particle dose distributions sensitive to targeting uncertainties. Purpose of this work was to investigate the robustness of scanned-beam particle therapy plans against setup errors for different optimization modalities, beam setups and ion species.

**Material and methods:**

For 15 patients with skull base tumors, localized in regions of severe tissue density heterogeneity, scanned lateral-opposed-beam treatment plans were prepared with the treatment planning system TRiP98, employing different optimization settings (single- and multiple-field modulation) and ion species (carbon ions and protons). For 10 of the patients, additional plans were prepared with individually selected beam setups, aiming at avoiding severe tissue heterogeneities. Subsequently, multiple rigid positioning errors of magnitude 1–2 mm (i.e. within planning target expansion) were simulated by introducing a shift of the irradiation fields with respect to the computed tomography (CT) data and recomputing the plans.

**Results:**

In presence of shifts, in carbon ion plans using a lateral-opposed beam setup and fulfilling clinical healthy tissue dose constraints, the median reduction in CTV V_95*%*_ was up to 0.7 percentage points (pp) and 3.5 pp, for shifts of magnitude 1 mm and 2 mm respectively, however, in individual cases, the reduction reached 5.1 pp and 9.7 pp. In the corresponding proton plans similar median CTV V_95*%*_ reductions of up to 0.9 pp (1 mm error) and 3.4 pp (2 mm error) were observed, with respective individual-case reductions of at most 3.2 pp and 11.7 pp. Unconstrained plans offered slightly higher coverage values, while no relevant differences were observed between different field modulation methods. Individually selected beam setups had a visible dosimetric advantage over lateral-opposed beams, for both particle species. While carbons provided more conformal plans and generally more advantageous absolute dose values, in presence of setup errors, protons showed greater dosimetric stability, in most of the investigated scenarios.

**Conclusion:**

Residual patient setup errors may lead to substantial dose perturbation in scanned-beam particle therapy of skull base tumors, which cannot be dealt with by planning target expansion alone. Choice of irradiation directions avoiding extreme density heterogeneities can improve plan stability against such delivery-time uncertainties.

## Background

The so-called inverse depth-dose profile and the sharp lateral penumbra make charged particles, like protons and carbon ions, apt for high-precision dose-escalated radiotherapy, e.g. in the treatment of intracranial tumors, due to the direct vicinity of critical structures [1,2]. Carbon ions offer additionally an enhanced relative biological effectiveness (RBE) [1].

Yet, based on physical properties of dose deposition by charged particle beams, it is expected that these advantages come to the price of greater sensitivity to delivery-time targeting uncertainties as both, the geometrical miss due to sharp lateral gradients and the displacement of the density heterogeneity interfaces within the treatment field, may result in delivered dose deterioration, which may not be completely dealt with by planning target expansion [3]. For scanned-beam intensity-modulated treatments, which can produce steep dose gradients and often rely on them to achieve their superior conformity, the problem of dosimetric robustness is likely of greater concern [3]. In this respect, a role may also be played by the physical dose modulation typically present in biologically-optimized carbon ion treatment plans, on account of the variable RBE, depending on multiple factors, including local dose [4].

The importance of the selection of an optimal plan, not only in terms of clinical dosimetric quality (target dose coverage versus normal tissue sparing), but also taking into account robustness, i.e. degree of sensitivity of the plan to the uncertainties involved in the treatment process, has been recognized by the ICRU [5] and emphasized, more recently, by Cantone *et al.* [6]. One method to assess the relevance of the above-mentioned dosimetric effects and to support clinical decision making is recomputation of treatment plans in geometries simulating the expected errors. Jäkel *et al.* [7] reported this procedure as part of patient-specific quality assurance for scanned carbon ion therapy, while a method for the evaluation of such tests was demonstrated by Lomax [3].

While the dosimetric robustness of particle therapy treatment plans has been extensively studied for other indications, e.g. prostate tumors [8,9], the published literature on cranial tumors is sparse. Recently, Hopfgartner *et al.* [10] investigated the robustness of proton treatment plans with respect to interfractional setup uncertainties, while the only previous work addressing this issue for carbon ions, a conference communication by Ellerbrock *et al.* [11], focused primarily on the evaluation of the multiple-field optimization approach, i.e. full intensity modulation (typically referred to as IMPT), as opposed to the single-field uniform-dose (SFUD) approach.

In this study we apply extensive plan recomputation, on a cohort of patients with skull base tumors, in order to systematically assess dosimetric robustness of scanned beam charged particle treatment plans in presence of setup errors and critical heterogeneities for (1) various plan optimization modalities, (2) different beam setups and (3) two particle species (carbon ions and protons).

## Material and methods

### Patient data

The data of 15 patients with skull base tumors (P1–P15) treated at our institution with intensity-modulated radiotherapy (IMRT) (P1, P13) or with stereotactic radiotherapy (SRT) (remaining) were chosen, with tumor localization typical for particle therapy indications (chordoma, chondrosarcoma, adenocystic carcinoma), in areas of high density heterogeneity and close to dose limiting structures.

Planning computed tomographies (CT) were acquired, without contrast medium, with in-slice resolution and slice thickness of 0.98 mm/pixel and 3 mm (P1, P13), 0.59 mm/pixel and 3 mm (P2, P11–P12) and 0.59 mm/pixel and 2.5 mm (remaining). The original clinical target volume (CTV) and organs at risk (OAR) contours, retained for the study, were delineated in the Pinnacle3 (Philips Healthcare, Best, The Netherlands) treatment planning system (TPS) for IMRT cases and in the Virtuos (Stryker-Leibinger, Freiburg, Germany) system for SRT cases, excluding unnecessary air cavities from target volumes. The median (range) CTV volume was 45.2 (15.6–90.7) cm^3^. The planning target volume (PTV) was defined, similarly to Orecchia *et al.* [2], by applying a margin of 2 mm in anteroposterior (AP) and mediolateral (LR) direction and of 2.5–3 mm in superoinferior (SI) direction, depending on CT slice thickness. The CTV-to-PTV expansion was performed on a single TPS to avoid potential inter-system algorithm variability. A contour separating the skin surface from the immobilization mask was added and densities outside it overridden with air, to exclude mask-specific heterogeneities from the investigation.

Following our institutional ethics procedures, all patients were informed of potential retrospective research use of their data and given the choice to opt out. The data presented in this manuscript do not reveal, either directly or indirectly, the patients’ identities.

### Treatment planning

Carbon ion and proton treatment plans for raster-scanning delivery [12] were prepared using the TRiP98 planning system (GSI Helmholtzzentrum für Schwerionenforschung, Darmstadt, Germany) [4,13]. The conversion program *dcm2trip*, developed at our institution and distributed with TRiP98, was used to convert the DICOM data (CT images and structure sets) to the VOXELPLAN format used by TRiP98 [14].

The local effect model LEM I was used for calculation of the RBE of carbon ions (*α*/*β*=2 Gy, threshold dose D_t_=30 Gy, nuclear radius r=4.5*μ*m), while a fixed value of 1.1 was employed for proton RBE.

The prescription dose was set to 60–63 Gy(RBE) delivered in 20–21 fractions for carbon ion plans and 64–74 Gy(RBE) in 32–37 fractions for proton plans similarly to Nikoghosyan *et al.* [15,16] and to Ares *et al.* [17]. The planning objective was to deliver at least 95% of the prescription dose to 95% of the PTV (V_95*%*_≥95*%*) and at least 95% of the prescription dose to 98% of the CTV volume (V_95*%*_≥98*%*), if possible without compromising organs at risk.

Also OAR dose constraints were derived from the protocols adopted in the above-mentioned studies. A maximum dose (D_max_) constraint at 54 Gy(RBE) was defined for optic nerves, optic chiasm and brainstem, however, the brainstem surface (1% of volume) abutting the PTV was allowed to receive a dose of up to 60 Gy(RBE) [15,16]. In proton plans, the corresponding limits were 60 Gy(RBE) and 63 Gy(RBE) [17].

For each patient two carbon ion treatment plans and a proton plan were prepared using two lateral-opposed beams (LR), reflecting the geometry typically available at fixed-nozzle carbon ion facilities.

The two carbon ion plans had, respectively, OAR constraints requiring per-field modulation (single-field optimization, denoted as *sc*) and full plan modulation (multiple field optimization, denoted as *mc*). The latter modality was also used in proton planning. Additionally, for comparison, plans without OAR constraints (unconstrained mode, denoted as *uc*) were prepared for both ion species.

Finally, for selected patients (n=10), carbon ion and proton plans using full modulation (*mc*) were prepared using two beams with individually selected directions. By visually analyzing potential beam directions, those were selected that appeared to minimize the presence of density interfaces along the entrance channels, that could introduce range variations in case of setup errors. These beam setups, referred to as *robust* (ROB) in the remainder of the text, employ a range of isocentric couch rotations (yaw) towards cranial direction, combined, in the majority of cases, with beam inclination about the longitudinal axis. Such gantry-rotation-like setups are enabled, at our facility, by combined use of 45-degree-inclined beam lines, 6-degree-of-freedom robotic couches (enabling patient roll and pitch up to 15 degrees with proper immobilization) and, when required, individualized solutions (immobilization with an advantageous head rotation or treatment in prone position).

At synchrotron based facilities, the available pencil beam transversal widths (spot sizes) in air at the treatment room isocenter are a function of the beam energy [13]. For carbon ion plans pencil beam full-width at half-maximum varied from 7.5 mm to 5.0 mm, as specified for the required energy range in the TPS synchrotron library, compatible with the GSI facility during the German ion beam therapy pilot project and representative of modern combined-beam facilities, like the Particle Therapy Center in Marburg. For proton plans spot sizes between 11.0 mm and 5.5 mm were enabled by treatment planning and delivery with shorter nozzle-to-patient distance than for carbon ions [18]. These planning settings enabled the highest available comparability of treatment plans, as they resulted in proton spot sizes, measured at patient surface, as close as possible to the corresponding ones of carbon ions.

In the irradiation raster, the transversal distance between neighboring spots (pitch) was set to 2 mm for carbon ion plans and 3 mm for proton plans. A tolerance of 0.4–1.0 times the spot size was set, allowing the TPS to place additional raster spots outside the target volume projection, transversely to the beam, to ensure PTV coverage without the need for high fluence spots at the target border. For carbon ion plans, a ripple filter was used to broaden the narrow pristine Bragg peaks and enable an in-depth peak positioning step of 3 mm [13]. For proton planning an in-depth peak distance of 2 mm was used. In the plans of one patient (P03) a bolus of 20 mm of water-equivalent material was used to ensure coverage of a tumor section shallower than the penetration depth of the lowest energy available. The dose grid resolution was the same of the planning CT.

### Dosimetric robustness tests

Rigid positioning setup errors were simulated by introducing shifts of the irradiation fields with respect to the CT data and recomputing the dose without plan reoptimization.

All plans were tested against 52 shifts, divided in two groups respectively of 3D magnitude 1 mm and 2 mm. For each magnitude the group represents a complete enumeration of shifts with components, in the patient’s coordinate system (LR, AP, SI), either zero or equal in absolute value. In the figures, individual shifts are expressed through their magnitude and a triplet, with the + and - signs indicating the direction of the (equal) non-zero components. The following formula generates the set S of all 52 tested shifts, expressed in mm, in the patient’s coordinate system:

$$\begin{array}{@{}rcl@{}} S &=& \left\{\vphantom{\sqrt{x^{2}+y^{2}+z^{2}}} \: (x,y,z) \: | \: \exists c \in \mathbf{R}, \: c > 0, \: x,y,z \in \{ 0, \pm{} c \},\right.\\ &&\;\;\left.\sqrt{x^{2}+y^{2}+z^{2}} \in \{1, 2\} \: \right\} \end{array} $$

### Data evaluation

The treatment plans were compared in terms of dose distribution and dose-volume histograms (DVH). For a quantitative assessment of the dose distribution in the PTV and CTV the 95% isodose coverage index (V_95*%*_) and the homogeneity index (HI), calculated as the difference (D_2*%*_ – D_98*%*_), were used [5], along with the PTV conformity index (CI) [19]. To evaluate OAR dose, the maximum dose (D_max_) was reported for the optic structures and the near-maximum dose at 1% volume (D_near-max_) for the brainstem. Visual and quantitative dose distribution analysis was entirely performed with *trip2png* [20], developed at our institution.

To analyze dosimetric plan robustness, the differences of the index values between the recomputed (shifted) and the initial plan were employed and denoted in the following as *Δ*V_95*%*_, *Δ*HI and *Δ*D_(near-)max_ respectively.

For selected comparisons and indices, statistical significance was assessed through a non-parametric, paired-sample sign test with a significance level of 0.05, using the R environment for statistical computing [21].

## Results

### Initial dosimetric quality of treatment plans

The mean (range) prescribed dose was 61.8 (60–63) Gy(RBE) for carbon ion plans and 66.9 (64–74) Gy(RBE) for proton plans. The initial (no shift) plans showed comparable target coverage for all planning approaches. The median PTV and CTV V_95*%*_, PTV and CTV HI, PTV CI and OAR D_(near-)max_ values for all planning scenarios are shown in Table 1. In all carbon ion treatment plans, the planning objective of PTV V_95*%*_≥95.0*%* was fulfilled, except in one of the *sc*LR plans, where it was compromised to 93.1% in order to meet OAR sparing constraints. For the same reason, for protons the planning objective (PTV V_95*%*_≥95.0*%*) had to be compromised twice in *mc*LR plans (to 94.4% and 90.4%) and once in *mc*ROB plans (to 93.1%).

**Table 1:** **Selected median (range) dosimetric indices of the optimized plans**

**Planning approach**									
**Dosimetric index**		**Carbon ion plans**					**Proton plans**		
		***uc*** **LR**	***sc*** **LR**	***mc*** **LR**	***mc*** **ROB**		***uc*** **LR**	***mc*** **LR**	***mc*** **ROB**
PTV V_95*%*_		99.2	97.9	98.3	98.3		97.2	95.2	95.8
[*%*]		(98.3-99.7)	(93.1-99.1)	(95.0-99.3)	(95.2-98.7)		(96.0-99.3)	(90.4-97.7)	(93.1-97.3)
CTV V_95*%*_		100.0	99.7	99.8	99.7		99.5	98.8	99.2
[*%*]		(99.9-100.0)	(97.3-100.0)	(98.5-100.0)	(98.8-100.0)		(99.2-99.8)	(95.5-99.6)	(97.8-100.0)
PTV HI		5.9	7.8	7.7	7.8		11.2	13.9	12.5
[*p* *p*]		(4.5-8.1)	(6.0-11.1)	(5.5-10.3)	(6.6-10.8)		(6.3-12.9)	(9.9-14.9)	(9.5-13.8)
CTV HI		4.0	4.8	4.6	5.0		7.9	9.4	8.7
[*p* *p*]		(3.4-5.0)	(3.6-8.2)	(3.7-7.7)	(4.5-7.3)		(5.1-9.3)	(7.0-12.5)	(6.3-9.9)
PTV CI		0.85	0.87	0.87	0.86		0.78	0.79	0.78
[−]		(0.81-0.91)	(0.80-0.91)	(0.82-0.91)	(0.82-0.89)		(0.70-0.88)	(0.71-0.87)	(0.71-0.84)
D_near-max_ brainstem		n/a	53.9	52.9	52.2		n/a	61.0	60.2
[Gy(RBE) ]			(38.8-55.0)	(35.6-54.2)	(36.9-54.3)			(45.5-63.1)	(46.3-62.6)
D_max_ chiasm		n/a	54.0	53.9	53.8		n/a	58.1	59.9
[Gy(RBE) ]			(0.0-54.2)	(0.0-54.1)	(52.6-54.1)			(0.0-60.1)	(59.0-60.2)
D_max_ ips. optic nerve		n/a	54.0	53.8	53.8		n/a	59.7	60.1
[Gy(RBE) ]			(0.0-54.2)	(0.0-54.2)	(40.6-54.1)			(0.0-60.3)	(50.9-60.4)
D_max_ con. optic nerve		n/a	42.9	43.3	39.2		n/a	51.2	42.5
[Gy(RBE) ]			(0.0-54.2)	(0.0-54.2)	(0.0-53.9)			(0.0-60.5)	(0.0-59.8)

*Abbreviations:* *CTV* clinical target volume, *PTV* planning target volume, *ips* ipsilateral, *con* contralateral, *LR* lateral-opposed beam setup, *ROB* robust beam setup, *uc* unconstrained mode, *sc* single-field modulation, *mc* multiple-field modulation, *pp* percentage point, *n/a* not applicable.

### Plan robustness

**Figure 1:**
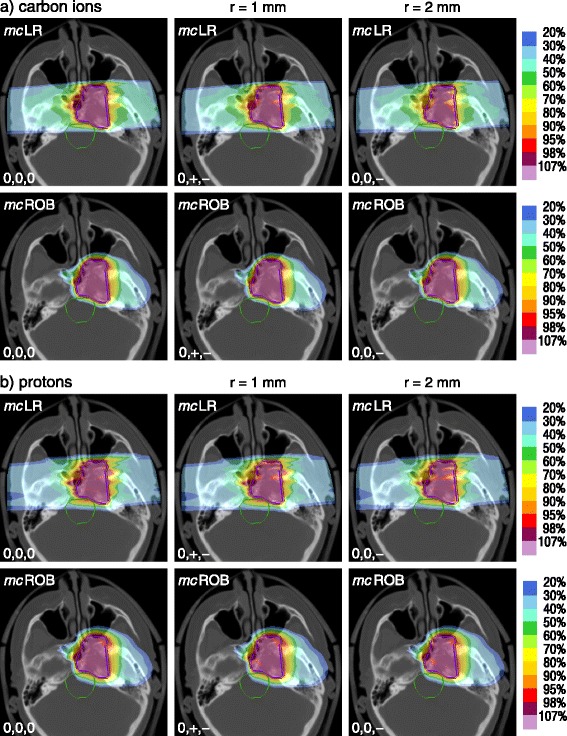
**Examples of setup error effects on dose distributions.** For an example patient, **(a)** carbon ion and **(b)** proton dose distributions of two planning approaches (*mc*LR and *mc*ROB) as planned (0,0,0) and re-computed in presence of positioning errors of 1 mm (0,+,-) and 2 mm (0,0,-). In the shifts + and - indicate the direction of (equal) non-zero components. *Abbreviations:* LR – lateral-opposed beam setup, ROB – robust beam setup, *mc* – multiple-field modulation.

Figure 1 presents carbon ion and proton axial dose distributions of an example patient, as planned and as resulting from exposure to setup errors of magnitude 1 mm and 2 mm, for various planning scenarios: a plan with multiple-field optimization and lateral-opposed beam directions (*mc*LR) and a plan with multiple-field optimization and individually chosen, robust beam directions (*mc*ROB). The lateral-opposed-beam dose distributions for both particle species show cold spots reaching into the CTV volume, despite the 2 mm CTV-to-PTV margin, a typical consequence of particle beam displacement with respect to a strong tissue density interface. The underdosage is not present in dose distributions from robust beam setups.

**Figure 2:**
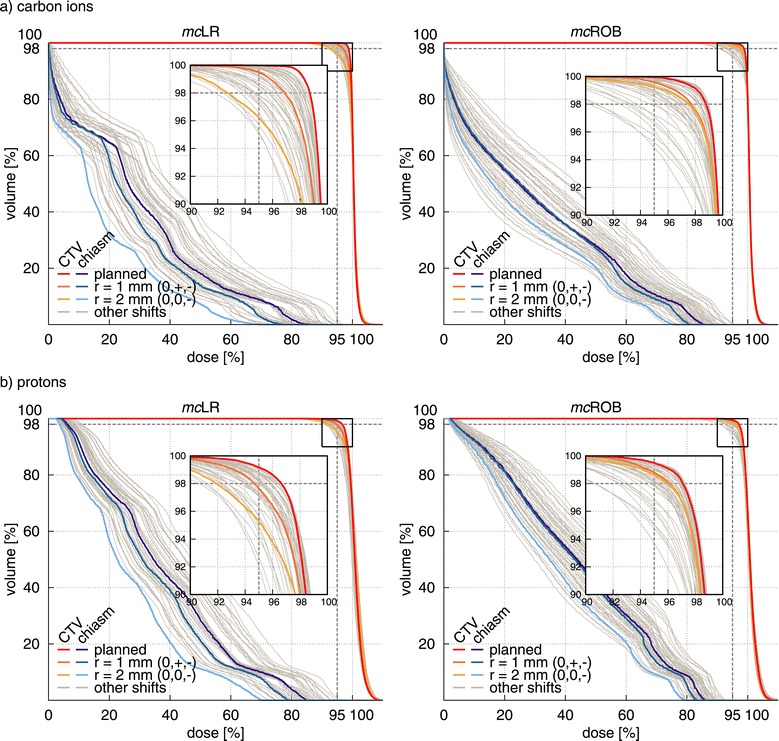
**Examples of setup error effects on dose-volume histograms (DVH).** For the example patient in Figure 1, CTV and chiasm DVH of **(a)** carbon ion and **(b)** proton treatment plans using two different planning approaches (*mc*LR and *mc*ROB) as planned and re-computed in presence of positioning errors. In color the original plan and two setup errors, of 1 mm (0,+,-) and 2 mm (0,0,-), shown in Figure 1. In grey all other simulated setup errors. In the shifts, + and - indicate the direction of (equal) non-zero components. *Abbreviations:* CTV – clinical target volume, LR – lateral-opposed beam setup, ROB – robust beam setup, *mc* – multiple-field modulation.

The corresponding dose-volume histograms of CTV and chiasm are presented in Figure 2, together with the DVHs of all other simulated shifts, illustrating the range of target coverage reduction and OAR involvement variability.

**Figure 3:**
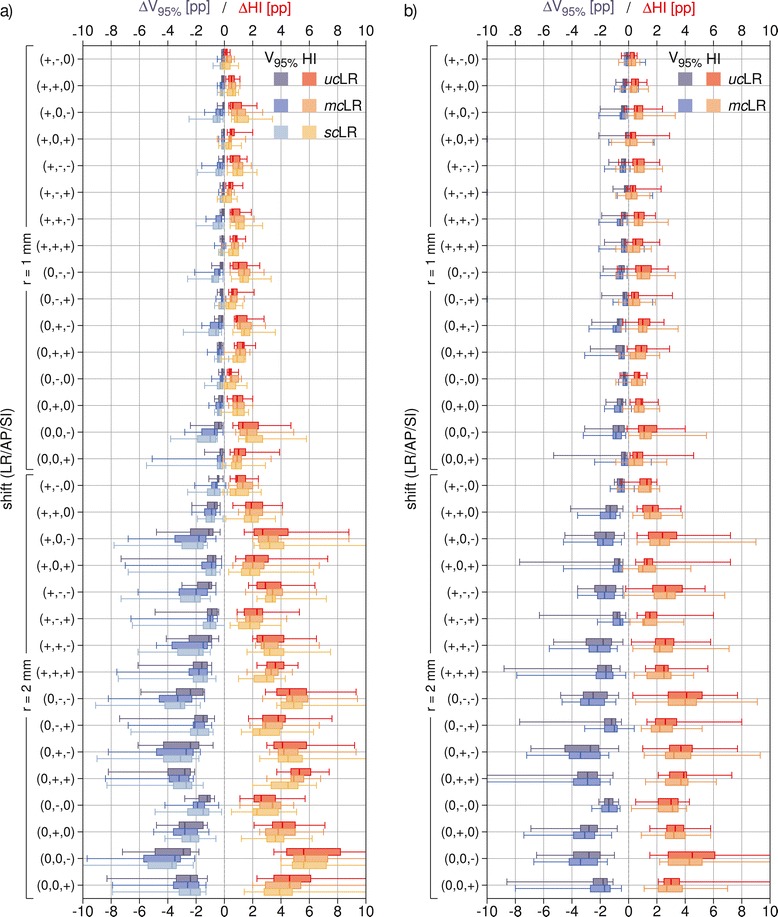
**Population box-and-whiskers plots of CTV dose coverage and homogeneity with detailed comparison of optimization modalities.** Population **(a)** carbon ion and **(b)** proton median value (within box), 1^st^ and 3^rd^ quartiles (box) and minimum and maximum values (whiskers) of CTV *Δ*V_95*%*_ and *Δ*HI for selected setup errors on LR plans with different optimization modalities: *uc*, *mc* and *sc*. In the shifts + and - indicate the direction of (equal) non-zero components. *Abbreviations:* CTV – clinical target volume, LR – lateral-opposed beam setup, *uc* – unconstrained mode, *sc* – single-field modulation, *mc* – multiple-field modulation.

The changes of carbon ion and proton CTV V_95*%*_ and HI induced by selected shifts, across the studied patient cohort, are shown in Figure 3. Since a shift along the beam does not introduce any density variation, i.e. any dose perturbation, for each pair of setup errors differing only in the sign of the LR component, only one is shown (positive LR component), for the sake of figure readability.

In general, setup errors caused a reduction of target coverage and homogeneity in the dose distributions. For lateral-opposed carbon ion plans making use of beam modulation (*mc*LR), the median reduction in CTV V_95*%*_ was up to 0.7 pp and 3.5 pp, for the worst-case shifts of magnitude 1 mm and 2 mm respectively. In individual cases, notably stronger coverage deterioration has been observed, with CTV V_95*%*_ decreasing by up to 5.1 pp and 9.7 pp respectively. The corresponding values for the proton plans of the same type were comparable, with median CTV V_95*%*_ reduction of at most 0.9 pp (1 mm error) and 3.4 pp (2 mm error) and respective individual case reduction of at most 3.2 pp and 11.7 pp.

**Figure 4:**
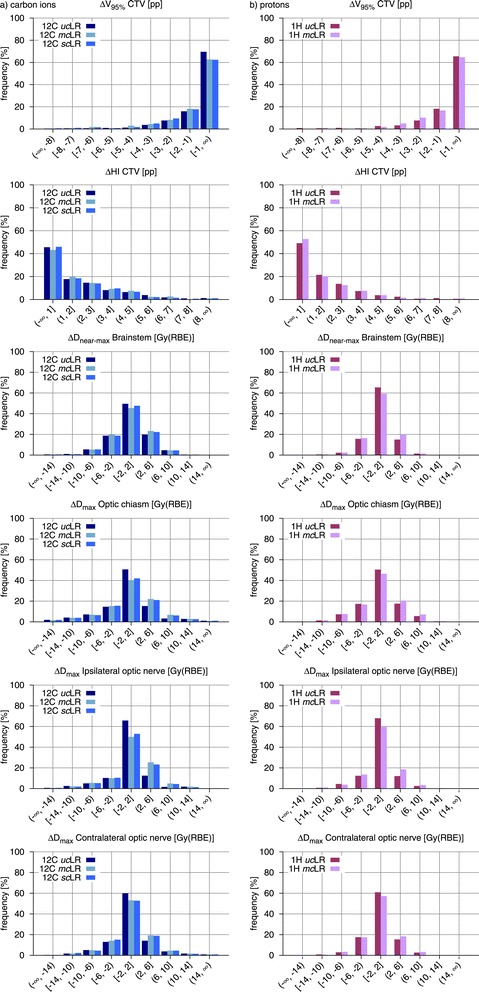
**Population frequency histograms of variations in dosimetric indices for all simulated setup errors and different optimization modalities.** Frequency histograms of the observed CTV *Δ*V_95*%*_, CTV *Δ*HI and OAR *Δ*D_(near-)max_ comparing LR treatment plans with different optimization modalities (*uc*, *mc* and *sc*) for **(a)** carbon ions and **(b)** protons. *Abbreviations:* CTV – clinical target volume, OAR – organ(s) at risk, LR – lateral-opposed beam setup, *uc* – unconstrained mode, *sc* – single-field modulation, *mc* – multiple-field modulation.

**Figure 5:**
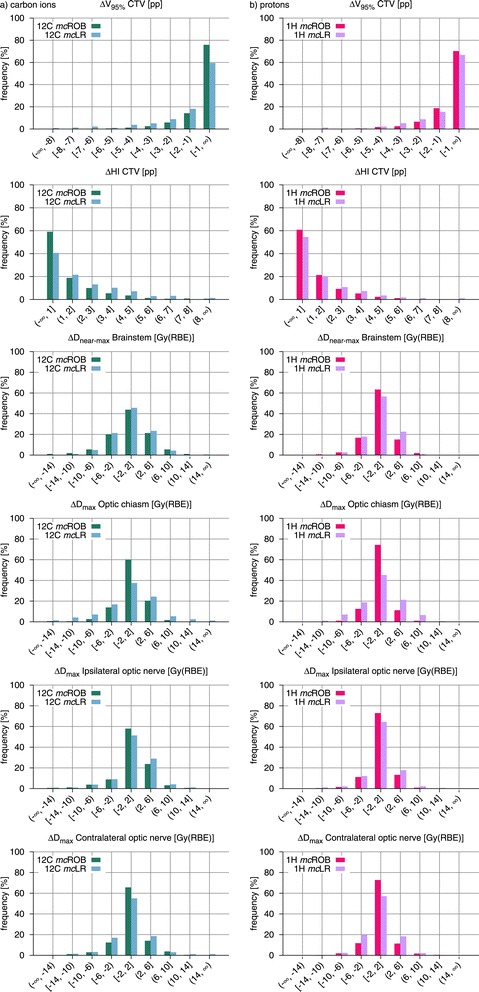
**Population frequency histograms of variations in dosimetric indices for all simulated setup errors and different beam setups.** Frequency histograms of the observed CTV *Δ*V_95*%*_, CTV *Δ*HI and OAR *Δ*D_(near-)max_ comparing *mc* treatment plans adopting LR and ROB beam setups for **(a)** carbon ions and **(b)** protons. *Abbreviations:* CTV – clinical target volume, OAR – organ(s) at risk, LR – lateral-opposed beam setup, ROB – robust beam setup, *mc* – multiple-field modulation.

**Figure 6:**
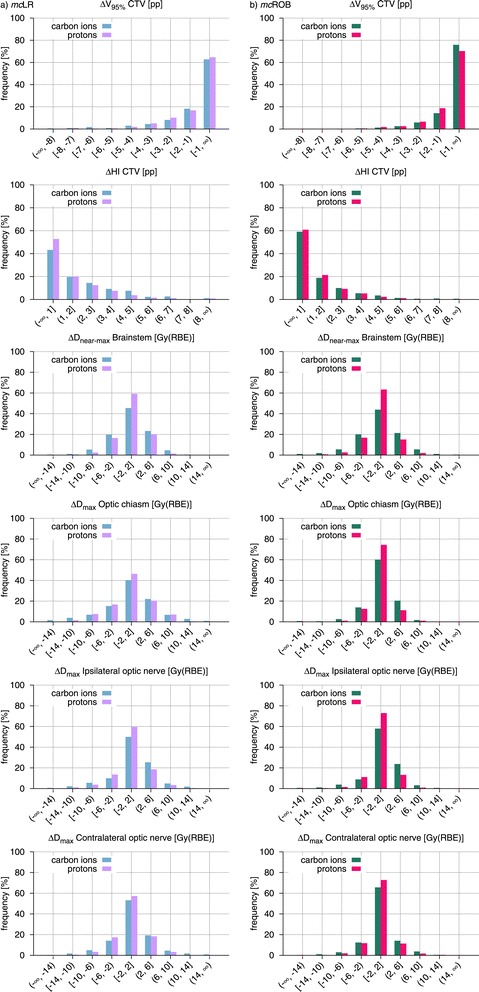
**Population frequency histograms of variations in dosimetric indices for all simulated setup errors and different ion species.** Frequency histograms of the observed CTV *Δ*V_95*%*_, CTV *Δ*HI and OAR *Δ*D_(near-)max_ comparing carbon ion and proton treatment plans with **(a)** *mc*LR and **(b)** *mc*ROB planning approaches. *Abbreviations:* CTV – clinical target volume, OAR – organ(s) at risk, LR – lateral-opposed beam setup, ROB – robust beam setup, *mc* – multiple-field modulation.

In order to assess and compare the overall robustness of treatment plan groups, histograms of the observed CTV V_95*%*_, CTV HI and OAR D_(near-)max_ changes are presented in Figures 4, 5 and 6.

### Comparison of optimization modalities

#### Carbon ion plans

Carbon ion plans introducing OAR sparing constraints through full intensity-modulation (*mc*LR) exhibited, in presence of setup errors, higher losses in target coverage, in comparison to the unconstrained counterparts. As visible in Figure 3, these differences in CTV *Δ*V_95*%*_ were only slight in median value (within 1 pp), for all simulated shifts, while in individual cases they reached 3.7 pp. This reduction of robustness, induced by the modulation of the irradiation fields, is also reflected in the histograms in Figure 4, with the predominance of smaller CTV V_95*%*_ changes in the unconstrained plans (about 70% vs. 63% of displacements resulting in *Δ*V_95*%*_ smaller than 1 pp in absolute value). Additionally, in about half of the simulated shifts, the reduction of CTV V_95*%*_, induced by the additional field modulation, was found to be statistically significant, although the generally low differences in median value suggest a lack of clinical relevance.

The effect of setup errors on target dose homogeneity appeared to be virtually unaffected by the difference in field modulation. For all simulated shifts, median CTV *Δ*HI of *mc*LR plans oscillated about the corresponding *uc*LR values without a clear trend and always below 1 pp in absolute value. Also the CTV HI histograms in Figure 4 show no distinct effect.

Increased incidence of larger D_max_ changes in critical structures was also observed for constrained plans (Figure 4). In particular, for the ipsilateral optic nerve in 16% more of the cases the D_max_ change exceeded 2 Gy(RBE) in absolute value. For the contralateral optic nerve this difference was 6% and for optic chiasm 10%. The increase in beam modulation almost did not affect D_near-max_ stability of the brainstem: the number of observations with an absolute deviation larger than 2 Gy(RBE) varied by less than 4% of the total, between the two optimization approaches. In the majority of cases under investigation, the brainstem was abutting the PTV, with its surface located in the strong dose gradient region for all planning modalities, rendering D_near-max_ subject to significant changes for all plans.

Regarding *sc*LR treatment plans, even smaller differences between them and the corresponding *mc*LR were observed. Throughout all simulated shifts the variation in CTV *Δ*V_95*%*_ was at most 0.4 pp in median value and 1.3 pp in single cases. Also the smaller deviations in the histograms (Figure 4) suggest that in practice no difference in robustness against setup errors exists between the two constrained planning approaches, at least at the levels of field modulation required for the indications under investigation.

#### Proton plans

For proton plans, the effect of modulation on CTV coverage, in presence of setup errors, was less pronounced and did not present a clear trend (Figure 3). Also for protons, median CTV V_95*%*_ was slightly better preserved in unconstrained plans, throughout the simulated shifts, but this was not always true in individual cases. Together with the small differences in median value, a paired-sample sign test reported, for almost all investigated shifts, no statistically significant difference between the two proton planning approaches. The similar behavior of *mu*LR and *mc*LR plans is also confirmed by the respective CTV *Δ*V_95*%*_ histograms in Figure 4.

Median values throughout all simulated setup errors seem to suggest a better HI preservation in the case of constrained plans (Figure 3). This is also somewhat visible in the histogram in Figure 4, where smaller CTV HI changes appear to be a little more common (4% of cases more) in constrained than in unconstrained plans.

With the introduction of modulation, also an incidence of larger D_(near-)max_ changes for critical structures was observed, which was mostly pronounced for the ipsilateral optic nerve (8% cases more exceeding 2 Gy(RBE) in absolute value) and less marked for the other optic nerve, for the optic chiasm and for the brainstem (respectively 4%, 5% and 5%).

### Comparison of beam setups

#### Carbon ion plans

Carbon ion treatment plans with beam setups selected to avoid severe density inhomogeneities (*mc*ROB) exhibited, in presence of setup errors, greater dosimetric stability than the corresponding lateral-opposed plans (*mc*LR), in terms of preservation of both target coverage and dose homogeneity. Among all simulated shifts, *mc*ROB plans were affected by CTV V_95*%*_ loss of at most 2.5 pp in median value, reaching 6.3 pp in individual cases, which represents a clear improvement in comparison with the *mc*LR plans of the same patient cohort, with respective values of 4 pp and 9.7 pp. Similarly, for the CTV HI, *mc*ROB plans presented better median value preservation (by up to 1.6 pp) and particularly strong worst case reduction, almost halving the difference.

While a direct shift-by-shift comparison between the plans is not applicable, as the influence of each setup error is specific to irradiation direction, the advantage of robust over lateral-opposed beam setups is visible in the histograms in Figure 5 as strong dominance of smaller changes (below 1 pp in absolute value) in CTV V_95*%*_ (16% of cases more) and CTV HI (18% of cases more) for *mc*ROB plans and in the prolonged tails toward larger variations for *mc*LR plans.

Also organs at risk benefitted from the greater dosimetric stability, afforded by robust beam selection, in presence of setup errors. Reduced incidence of larger D_max_ changes, in comparison with the lateral-opposed plans, was observed for most of the critical structures: the index deterioration remained below 2 Gy(RBE) in absolute value in 25% of the shifts more for the chiasm, 11% more for the contralateral optic nerve and 7% more for the ipsilateral optic nerve. Only for the brainstem, which for nearly all patients was in contact with the target dose gradient region, no considerable improvement in D_near-max_ stability was observed.

#### Proton plans

In presence of setup errors, proton *mc*ROB treatment plans achieved, similarly to the equivalent carbon ion ones, better preservation of target coverage and dose homogeneity in comparison to plans with a lateral-opposed beam setup. In *mc*ROB plans, median and worst-case CTV V_95*%*_ losses were 2.8 pp and 5.8 pp respectively, while in the corresponding *mc*LR plan they were 3.6 pp and 11.7 pp. HI median and worst-case variations were instead 3.4 pp and 6 pp for *mc*ROB plans and 3.9 pp and 14.2 pp for *mc*LR plans. The dosimetric advantage in target dose conservation through robust beam configurations is visible in the V_95*%*_ and HI deterioration histograms in Figure 5, where *mc*ROB exhibit a larger number of cases (respectively 7% and 8%) affected by smaller absolute changes.

Proton *mc*ROB treatment plans also offered improved OAR dose stability. In comparison to the corresponding lateral-opposed plans, in a higher number of cases D_(near-)max_ remained in absolute value within 2 Gy(RBE) for the contralateral optic nerve (16% of cases more), ipsilateral optic nerve (9%), optic chiasm (29%) and brainstem (7%).

### Comparison of ion species

The preceding subsections already report data on the investigated scenarios, for both carbon ions and protons. Some results of potential interest are better described through direct comparison of the two ion species, as presented in Figure 6, for *mc*LR and *mc*ROB plans, which both represent clinically valid treatment planning approaches.

With a lateral-opposed beam setup protons presented greater dosimetric stability in the target, a result that is consistent with the lower initial conformity of proton plans used in this study (Table 1). Use of robust beam setups reversed this situation, affording greater CTV V_95*%*_ reproducibility to carbon ions and making the two particle species practically equal in terms of HI preservation. This result shows that applying a planning technique aiming at reducing physical effects of setup errors, like selecting less inhomogeneous beam paths, brought greater benefit to the particle species with the most conformal dose distributions. For both *mc*LR and *mc*ROB plans, protons showed greater reproducibility of planned OAR dose, in presence of setup errors, with the greatest advantage for D_near-max_ in the brainstem, that is the OAR closest to the target dose gradient (Figure 6).

Finally, it should be emphasized that, while in terms of relative changes, between optimized plans and simulated delivery with setup errors, protons demonstrated greater dosimetric stability (i.e. a larger number of smaller changes) for the majority of investigated indices and plan types, in terms of absolute dose values carbon ion plans used for this study remained superior in terms of both median target coverage and OAR sparing (Table 1).

## Discussion

Systematic plan robustness tests, like those presented in this manuscript, are necessary to understand the magnitude of delivery-time dose uncertainties, in order to optimize treatment planning methodologies and also to ensure the selection of the best treatment for individual patients.

In fact, despite its heavy requirements in terms of time and computing resources, a procedure based on multiple plan recomputations, similar to the one used in our study, was reported by Jäckel *et al.* [7] as part of the clinical patient-specific quality assurance process, for scanned carbon ion therapy. An alternative measurement-based method was proposed by Albertini *et al.* [22], to assess plan robustness by recording the effects of setup (or range) uncertainties on a plan delivered to an anthropomorphic phantom, thus excluding the inaccuracies of analytical dose computation algorithms, typically employed in clinical planning systems. While this is certainly a considerable advantage, this approach is of limited applicability to carbon ions, for which only the underlying absorbed dose distribution deterioration can be measured. Furthermore, relying on the standardized phantom anatomy appears more suitable a method to compare the influence of optimization approaches or planning settings for typical situations, approximated by target volume definition in the phantom. Instead, as also shown in our study, significant variability between the patients may be present, stemming from the differences between individual target volumes and anatomical features, as well as their spatial relation, which emphasizes the importance of conducting robustness assessments for specific patients (or well-defined populations) in order to support clinical decision making.

Alternatively, accurate dose computation on real patient geometries can be achieved through Monte Carlo methods, a particularly interesting option for proton therapy, for which a dedicated high-performance implementation was recently demonstrated [23]. For carbon ions, instead, while novel integrated tools can simplify clinical applications [24,25], the prolonged computational times must be weighed against the expected precision gain, especially in large scale computations. For one of the cases presented in this manuscript, for instance, Monte Carlo was employed on a single treatment plan and a single recomputation, demonstrating slight discrepancies in highly-heterogeneous regions and absorbed-dose DVH differences in the order of 2% [25], which warrant more extensive future investigations.

A critical point, when investigating the dosimetric effects of setup errors, is selecting the range of errors to simulate. Two approaches have been proposed in the literature, simulating either a small number of extreme displacements, representing the worst-case scenarios, or calculating numerous dose distributions, reflecting the entire possible range (and probability) of displacements. Data obtained in our study suggests that the former may lead to false conclusions, as the worst case is not known a priori and not always strictly correlated to the displacements magnitude. More importantly, the worst-case displacement is not the same for different beam setups, rendering direct comparisons of limited value. An example of this is the comparison of a lateral-opposed beam setup against other beam configurations in presence of mediolateral shifts, by which the former remains unaffected.

Therefore, the study presented in this manuscript was based on massive plan recomputation, with real patient data and a comprehensive, fine-grained set of simulated setup errors, and is, to our best knowledge, the first of this kind investigating multiple aspects, that affect carbon ion and proton dosimetric robustness, in skull base radiotherapy, on a multi-patient cohort.

The only previous study addressing the issue of robustness against setup errors for carbon ion plans, a conference communication by Ellerbrock *et al.* [11], reported an absolute reduction of the CTV V_90*%*_ by 0–1 pp for 1 mm displacements and 2–4 pp for 2 mm displacements in one patient. While this work, like ours, tested a broad range of errors, it focused primarily on usability of IMPT and employed plans with systematically varying weight of constraints, rather than clinically valid ones. It should also be noted that, to counterbalance the computation-time explosion of multiple optimizations, only absorbed dose computations were performed, neglecting both the additional field modulation and the potential compensation effects, introduced by variable carbon ion RBE. More recently, Hopfgartner *et al.* [10] systematically investigated the robustness of proton treatment plans with respect to interfraction setup uncertainties in 7 skull base (and 7 paranasal sinus) tumor cases for 6 extreme orthogonal displacements, reporting median CTV V_95*%*_ reductions of up to about 3 pp. In contrast to this study, which aimed at quantifying robustness in presence of extreme displacements for simple and more complex beam setups, selected with sole consideration of critical structure sparing, the goals of our work included extensive investigation of the robustness loss due to field modulation as well as its improvement through avoidance of critical heterogeneities in the beam channels.

Our results demonstrate that residual repositioning errors may, in some cases, lead to a significant perturbation of the delivered dose in scanned-beam carbon ion and proton therapy for skull base tumors (by up to 10 pp for carbon ions and up to 12 pp in proton plans, in individual cases), which cannot be completely dealt with by planning target expansion alone. Only rigid setup errors were simulated, as they represent the predominant source of targeting uncertainty in skull base treatments, with magnitudes (1–2 mm) consistent with the precision reported for modern non-invasive cranial immobilization devices and patient positioning systems, in terms of residual setup errors as well as intrafraction motion [2,26-30]. No rotational errors were simulated, as in comparison to translational displacements, angular deviations cause smaller effects as established in the preliminary work for this study [31] and also observed for other indications [8].

The clinical plans (fulfilling OAR constraints) displayed a slightly reduced robustness, understood as the spectrum of variation of dosimetric indexes induced by presence of simulated setup errors, as compared to plans prepared without OAR constraints, illustrating the isolated influence of the dose gradients within the individual, modulated fields. For individual shifts these differences however seldom reached statistical significance, confirming the observation by Ellerbrock *et al.* [11], that the generally stronger dose gradients at the field edge have more significant interactions with positioning uncertainties, while internal dose gradients, caused by inhomogeneous dose delivery by individual fields, are weaker and thus have minor influence on the robustness. Additionally, in carbon ion plans, only minimal differences were observed between the two modulation approaches (single- vs. multiple-field optimization). The strength of the planning constraints, which determines the magnitude of field modulation, is likely a factor influencing this result. In our study, typical dose modulation (defined here as the ratio between the maximum OAR dose and the prescription dose) was 0.85. The robustness of treatment plans where the application of stronger OAR constraints might be necessary, e.g. for re-irradiations, should be investigated for each individual case. Further complexity lies in the potentially large degeneracy of the optimization result. It has been demonstrated that dosimetrically equivalent IMPT plans employing different modulation strategies may be characterized by differing degrees of robustness [32].

One of the most interesting applications of robustness tests against an extensive set of setup errors is the direct comparison, in terms of dosimetric stability, of different beam-setups. In our study, for a subset of patients, improved plan robustness, with respect to both CTV and OAR dose, was demonstrated when using beam setups avoiding strongly heterogeneous tissue. It should be noted that the beam directions for this set of treatment plans were selected with sole consideration of the density homogeneity in the beam channels, i.e. not considering the position of critical structures, which were however spared through plan optimization (in compliance with the planning protocol).

Currently, clinical robustness testing of irradiation setups must be usually carried out by expert personnel, on a case-by-case basis, evaluating possible tradeoffs with planning constraints, like OAR sparing. This underlines the need for automatized tools to support such multi-criteria, optimal beam setup selection. Verification of the entrance channel homogeneity through pattern matching techniques, that mimic human analysis, has been demonstrated a promising approach, which can be easily combined, through an optimization process, with clinical constraints, like patient positioning limitations or OAR involvement [33,34]. Methods have also been proposed to include robustness in the plan optimization process in proton therapy [35] or in margin design [36,37], but they suffer from the limitation of being based on assumed distributions of positioning errors and do not have, up to now, widespread clinical application.

Finally, our study presents the first attempt to compare the dosimetric robustness of treatment plans employing different ion species. Between protons and carbon ions, used in our study, differences have been demonstrated in the basic dose deposition properties, that may be relevant in their application as therapeutic radiation types [38]. In our study, we strove to maintain proton and carbon ion plans as comparable as possible, in both technical (e.g. using similar raster spot sizes) and clinical terms (e.g. using comparable planning objectives). Also, for both particle species plans with two beams were used, as typical in carbon ion therapy of the skull base and not uncommon in proton therapy, especially at fixed-nozzle combined facilities [2,15,18]. Yet, in order to achieve clinically acceptable plans for both particle species, not all planning settings could be kept strictly equal, which should not be neglected when interpreting our results. For instance the significant beam broadening in matter undergone by protons required a larger target projection expansion, to achieve acceptable PTV coverage, and resulted in a lower initial conformity of the proton plans. While these were comparable, in terms of CTV coverage, to those investigated in a similar study by Hopfgartner *et al.* [10], it should be noted that, in our study, realistic proton spot sizes attainable at synchrotron-based combined ion therapy facilities were used, which were inferior to the idealized ones used by Hopfgartner *et al.* [10]. Additional proton beams, as often used when a rotating gantry is available, would have likely improved the initial conformity, but they would have also altered the robustness, as observed e.g. by Hopfgartner *et al.* [10].

Most notably, from a strictly numerical interpretation of our results, in most of the scenarios considered, in presence of setup errors protons appear to deviate less from the planned values, both in CTV coverage and OAR involvement, with a particular stability advantage for the brainstem, the organ closest to the target and often in direct contact with it. This observation could be of importance in clinical decision-making, e.g. when defining guidelines and thresholds for image-guided radiotherapy (IGRT) protocols, and is likely related to the more pronounced scattering protons undergo, resulting in typically shallower gradients. Another important observation, related to potential clinical applications, is that carbon ion plans, with their higher initial conformity and more advantageous absolute dosimetric values, received greater advantage, in terms of target dose reproducibility, through use of robust beam setups.

Ultimately, purpose of the comparison was not to identify the *best* particle species, but rather to demonstrate how the differences in the physical properties of ions, and in the clinical applications they enable, may affect our understanding of robustness issues. In this sense, a certainly valid general conclusion is that per-indication studies should be carried out for each ion species and some care should be used when translating results from one particle type to another. This is particularly important in the current context of combined facilities, where protons and carbon ions are used for different indications, and as newer ion species, e.g. oxygen or helium, are being considered for introduction in clinical particle therapy [39]. Similarly, where a radiobiological model is used to compute effective dose, results should not be considered valid across different models, model versions or model input parameters [40,41].

When assessing the impact of these results on patient treatments, one aspect, crucial for the interpretation of this study, should not be neglected. The application of the total therapeutic dose is typically fractionated, causing delivery uncertainties to be statistically smoothed, provided that they are not systematic. It is therefore advisable that, for a thorough clinical assessment, a similar study be conducted in the future, employing e.g. the results of daily positioning verification data, to assess cumulative dose. Finally, the positioning errors are only one of many potential delivery-time uncertainties that can affect the final dose distribution, like range uncertainties [32], anatomy changes [42], beam fluctuations [43], etc. For novel techniques, like carbon ion scanned-beam radiotherapy, the relevance of such uncertainties and of their interplay remains mostly to be quantified.
